# Senolytics for Cancer Therapy: Is All that Glitters Really Gold?

**DOI:** 10.3390/cancers13040723

**Published:** 2021-02-10

**Authors:** Valerie J. Carpenter, Tareq Saleh, David A. Gewirtz

**Affiliations:** 1 Department of Pharmacology and Toxicology and Medicine, School of Medicine, Virginia Commonwealth University, Richmond, VA 23298, USA; delucavj@vcu.edu; 2 Massey Cancer Center, Richmond, VA 23298, USA; 3 Department of Basic Medical Sciences, Faculty of Medicine, The Hashemite University, Zarqa 13133, Jordan; tareq@hu.edu.jo

**Keywords:** senolytic, cancer, senescence, chemotherapy, adjuvant, dormancy, recurrence, ABT-263, navitoclax

## Abstract

**Simple Summary:**

Senescence is an essential component of tumor cell biology and is a primary cell stress response to therapy. While the long-term impact of senescence in cancer therapy is not yet fully understood, the use of senolytics, drugs that selectively kill senescent cells, is an area of active investigation in cancer treatment. Several challenges and unanswered questions have arisen from the current preclinical literature, indicating the need to re-evaluate some of the basic premises and experimental approaches, as well as the potential utility for translating to the clinic the application of senolytics as adjuvants to current cancer therapy.

**Abstract:**

Senolytics represent a group of mechanistically diverse drugs that can eliminate senescent cells, both in tumors and in several aging-related pathologies. Consequently, senolytic use has been proposed as a potential adjuvant approach to improve the response to senescence-inducing conventional and targeted cancer therapies. Despite the unequivocal promise of senolytics, issues of universality, selectivity, resistance, and toxicity remain to be further clarified. In this review, we attempt to summarize and analyze the current preclinical literature involving the use of senolytics in senescent tumor cell models, and to propose tenable solutions and future directions to improve the understanding and use of this novel class of drugs.

## 1. Introduction

Within the last few years, senescence has been increasingly recognized as a central component of tumor biology and the response to anti-cancer therapies. In its simplest form, senescence is a stress response that occurs subsequent to replicative-, oxidative-, oncogene-, and therapy-induced insults [1,2,3,4]. Senescent cells undergo a prolonged growth arrest, yet remain metabolically viable, and can be identified by an array of phenotypes including structural changes [5,6], increased lysosomal biogenesis (senescence-associated beta galactosidase, SA-β-gal) [7,8], dysregulated metabolism [9,10], epigenetic changes [11], and extensive alterations in gene expression [12]. Senescence is also almost universally accompanied by the secretion of various soluble and insoluble factors, termed the senescence associated secretory phenotype (SASP) [13,14]. However, despite these hallmark features, it is important to understand that the senescent phenotype can be highly variable, based on cell type and senescence-inducing stimulus. For example, Hernandez-Segura et al. revealed a heterogeneity in senescence-associated gene expression in fibroblasts depending on whether senescence was induced by replicative exhaustion, oncogene hyperactivation, or ionizing radiation [15]. This same study also showed that the expression of key senescence-related genes was dynamic and could vary temporally [15]. Furthermore, the degradation of Lamin B1 is an established feature of senescence [16]; however, in oxidation-induced senescence, Lamin B1 may accumulate [17], providing another example of the phenotypic variability of senescence. The SASP is also heterogeneous, and its diverse composition based on cell type and stimuli has been well characterized by Basisty et al. [18]. Importantly, not all senescence-inducing stimuli will elicit a SASP; this is particularly the case for non-DNA damaging insults [19]. Finally, single cell sequencing of senescent cells has also revealed that this heterogeneity in gene expression can exist even within a monoclonal population of cells [20,21].

Premalignant and malignant (tumor) cells, although typically undergoing rapid replication, can also enter a senescent cell state, characterized by a stable growth arrest and the presence of multiple senescence hallmarks. During malignant transformation, for example, senescence can serve to delay or subvert the progression to tumorigenesis of premalignant cells, thereby acting as a tumor suppressive mechanism [22,23]. In established malignancies undergoing treatment, a plethora of anti-cancer drugs with variable mechanisms of action have been shown to promote a form of therapy-induced senescence (TIS) both in vitro and in vivo [24]. For example, conventional therapies such as etoposide, doxorubicin, and cisplatin are established inducers of TIS [25,26,27,28,29,30]. Targeted therapies, such as the BRAF inhibitor vemurafenib, EGFR inhibitor osimertinib, and hormonal therapies, including fulvestrant and androgen deprivation, have also been shown to result in senescence induction [31,32,33,34,35,36,37,38]. For an extensive review of drug classes that have been shown to promote TIS, we refer the reader to Saleh et al. [24]. Furthermore, human tumor samples from patients treated with genotoxic therapies have demonstrated that this response is also likely conserved clinically, although the evidence in support of this premise is more limited [25,39,40]. As such, it is highly probable that senescent tumor cells accumulate over time, both spontaneously and as a consequence of therapy.

While TIS has long been established in the cancer field, a full understanding of how senescence may impact patient outcome has been evolving and is far from complete. The long-held traditional paradigm argued that senescence was an irreversible cell fate [41], and as such, TIS was purported to be a beneficial outcome of therapy in that it could lead to permanent abrogation of established tumor growth [42,43]. However, in recent years, multiple reports have been generated in support of the premise that cells that have entered into TIS can, in fact, escape the senescent growth arrest; this has been shown following various therapies, including topoisomerase poisons [26,44,45], alkylating agents [46], ionizing radiation [47,48], cyclin-dependent kinase 4/6 inhibitors [49], PARP inhibitors [50], and androgen deprivation [35]. Furthermore, tumor cells that escape senescence have been reported to develop more aggressive phenotypes associated with increased stemness and drug resistance [51,52], although this is not universally the case, as some senescence escapers are only as neoplastic as their parental cells [53,54]. In addition, TIS in non-tumor cells has been linked with several untoward effects of cancer therapy, including cancer relapse [55], and more importantly, senescent tumor cells themselves have been demonstrated to directly account for the emergence of recurrent cancer phenotypes [56]. Therefore, while the senescent growth arrest may confer short-term advantages with regards to tumor progression, these beneficial effects may only be short-lived, and may be permissive for the development of more pernicious cancer phenotypes over an extended period of time.

The SASP is also double-edged. On the one hand, the SASP may activate the immune system and lead to tumor clearance [57]; on the other hand, the SASP can be immunosuppressive and may promote growth, epithelial-to-mesenchymal transition, and increased stemness in neighboring cells [58]. Consistent with these opposing functions, there is ample evidence showing that senescence can both contribute to deleterious effects of therapy, and conversely, be associated with better patient outcomes. These conflicting observations are likely explained by the heterogeneity of the senescent phenotype and the context in which it occurs (i.e., tumor type and treatment), emphasizing the critical need for a more in-depth understanding of TIS in the context of patient response to these therapeutic interventions [59].

In response to the identification of the potentially deleterious consequences of senescence, the development and characterization of small molecules that can modulate senescence has rapidly expanded. Senolytics, drugs that preferentially target senescent cells for cell death, were originally identified in studies of aging [60]. Of these agents, studies utilizing the BCL-X_L_/BCL-2 inhibitor navitoclax (ABT-263) or the dasatinib+quercetin (D+Q) combination have generated the most promising experimental data for the elimination of senescent cells. It is important to note, especially for compounds that influence multiple signaling pathways such as D+Q, the difference between senolytic and senomorphic activity. To be considered a senolytic, a compound must be capable of *killing* senescent cells. Alternatively, alteration of senescent phenotypes, such as the SASP, or reducing senescence induction without cell killing is more indicative of a senomorphic activity. Both ABT-263 and D+Q, for example, have been previously established to be senolytic, rather than senomorphic [60,61,62]. While senomorphics have been postulated to target the more unfavorable aspects of senescence, such as the inflammatory SASP [63], the use of senolytics has garnered more attention in the cancer field. These agents have now been favored by the cancer community largely under the premise that the combination—or “two-hit” approach—of senescence-inducing chemotherapies followed by senolytics may increase tumor cell killing and/or eliminate residual disease [64,65,66].

While the adoption of senolytics as adjuvant cancer therapeutics carries potential promise, multiple reports examining the effectiveness of different senolytic agents in combination with senescence-inducing therapies in cancer models have raised several issues and potential concerns. These include the lack of universality of senolytic action against different therapy-induced senescence models, the potential for systemic toxicity such as neutropenia and thrombocytopenia (in the case of ABT-263), the likelihood that resistance to the senolytics could develop, and their specificity for “harmful” senescent cells. In this review, we attempt to provide a critical assessment of current evidence in support of the utilization of senolytic therapy in cancer, some reservations relating to their clinical applicability, as well as to offer insight into how the senolytic strategy for cancer therapy might be optimized. We also propose that senolytics could be utilized initially for the purposes of reducing the likelihood of cancer recurrence based on the premise that their administration could promote the removal of senescent tumor cells that reflect a dormant population with the capacity for re-emergence and repopulation [67]. However, actually evaluating this possibility in the clinic would be extremely challenging, requiring extensive and lengthy monitoring periods, since tumor dormancy and disease recurrence is extremely variable and can occur over periods of months and possibly years. Consequently, this approach would likely be initiated, at first, in the types of cancer where current standards of care remain inadequate and which are associated with short patient-free survival periods.

## 2. Senolytic Therapies: Have We Hit Gold or Pyrite?

### 2.1. Established Success of Senolytic Therapy in the Mitigation of Aging-Associated Disease

The first step in the development of senolytics was the identification of transcriptomic signatures unique to irradiated, senescent human fat cell progenitors, or pre-adipocytes [60]. These cells showed a differential increase in pro-survival or anti-apoptotic pathways, consistent with the prolonged survival and persistence of senescent cells. Consequently, the targeted siRNA blockade of molecular targets involved in these pathways, including PI3K, p21^Cip1^, BCL-X_L_, or PAI-2, resulted in the killing of senescent cells but not their proliferating or quiescent counterparts [60]. This paved the way for the development of small molecule inhibitors targeting essential survival pathways in senescent cells. Via the screening of 46 potential agents, the first efficacious senolytic strategy utilized the combination of dasatinib+quercetin (D+Q) [60]. Dasatinib is a tyrosine kinase inhibitor that interferes with ephrin-dependent survival, whereas quercetin is a flavanol that inhibits various kinases, including PI3K [60]. The D+Q combination has been shown to clear senescent cells in vitro and in vivo in a variety of senescence or aging-related models including chronic atherosclerotic vascular disease [68,69], radiation- or bleomycin-induced lung fibrosis [70], aging-associated hepatic steatosis [71], arteriovenous fistulation in chronic kidney disease [72], Alzheimer’s disease [73,74], hyperoxia-induced airway dysfunction [75], obesity-associated anxiety [76], obesity-induced metabolic dysfunction [77], osteoarthritis [78], radiation-induced bone degeneration [79] and radiation-induced ulceration [80]. D+Q has been shown to ameliorate various organ system dysfunctions associated with these disorders, improve physical dysfunction and increase survival of naturally or experimentally-induced aged mice [81]. Moreover, D+Q is being investigated in clinical trials targeting chronic kidney disease, pulmonary fibrosis and Alzheimer’s disease [NCT02848131, NCT02874989, NCT04210986]. Early results have shown that D+Q can reduce senescent cell burden in vivo [82]. Moreover, D+Q improved physical function in 13 patients with idiopathic lung fibrosis despite unremarkable changes in senescence-associated biomarkers [83].

Another frequently utilized senolytic is the BH3 mimetic, ABT-263 (navitoclax). ABT-263 was first tested as a senolytic based on the dependence of some senescent cells on the pro-survival proteins of the BCL-2 family [84]. As is the case for other BH3 mimetics, ABT-263 promotes apoptosis by binding pro-survival BCL-2 family members and preventing their association with pro-death BCL-2 family members [85]. The functional activities of BCL-2, BCL-X_L_, and BCL-W, but not MCL-1, are inhibited by ABT-263 [85]. Originally conceived as a cancer therapeutic, ABT-263 was synthesized using structure-based drug design and was later found to selectively induce apoptosis in senescent bone marrow hematopoietic stem cells, which resulted in bone marrow recovery following total body irradiation in mice [62]. Notably, the senolytic action of ABT-263 appears to depend on inhibition of BCL-X_L_ and/or BCL-W, while BCL-2 inhibition is dispensable [84,86]. Similar to D+Q, ABT-263 was successful in eliminating senescent cell populations in several disease models including aging-associated bone loss [87], radiation-induced lung fibrosis [88], lung emphysema [89], uterine leiomyoma [90], tau-dependent neurodegenerative disease [91], radiation-induced neurodegeneration [92], myocardial infarction (including ischemia-reperfusion injury) [93,94], heart failure [95], pulmonary hypertension [96], insulin resistance [97], osteoarthritis [98,99], synthetic implant-mediated fibrosis [100], and Duchenne muscular dystrophy [101]. ABT-263 is currently a cornerstone in preclinical studies of senolysis and remains a promising drug for use against both liquid and solid tumors [102,103,104].

Both dasatinib and navitoclax are synthetic small molecules that were initially developed as cancer therapeutics but are now under consideration for the mitigation of aging-related pathologies. Other naturally occurring substances with potential senolytic activity and low overall toxicity are also under investigation. In addition to quercetin, fisetin has also shown senolytic activity. Fisetin belongs to the flavonoid family, which are a group of natural chemicals widely available in plants, including several vegetables and fruits. Of 10 flavonoids tested, fisetin showed the greatest efficacy in eliminating subpopulations of senescent mouse and human adipose cells; fisetin increased both the median and maximum lifespan of mice, accompanied by a significant reduction of senescence-associated markers [105]. Fisetin also promoted the killing of senescent human umbilical vein endothelial cells (HUVECs) but not senescent IMR90 human lung fibroblasts or senescent primary human preadipocytes, reflecting a somewhat narrow-spectrum and cell type-specific activity [106]. Piperlongumine is a natural product found in the fruit of Piper longum that was also identified, through drug screening, to exert senolytic activity in radiation-, replicative- and oncogene-induced senescent WI-38 fibroblasts [107]. Both curcumin, a primary component in turmeric, and its synthetic analogue EF24, exhibit senolytic activity [108,109]. A number of additional compounds have been found to exhibit a range of senolytic effects, with the majority being established drugs with newly discovered ability to kill senescent cells and contribute to the amelioration of aging-related pathologies (reviewed in [24]).

### 2.2. The Dual Faces of Therapy-Induced Senescence

A fundamental premise underlying the use of senolytic agents as adjuvant to cancer therapy is that the accumulation of senescent tumor cells following exposure to conventional or targeted chemotherapeutics is deleterious. Since the first report to demonstrate TIS [110], it was anticipated that the induction of senescence in tumor cells would impose an irreversible growth arrest, thereby indefinitely suppressing tumor progression [111,112], supporting the development of agents that are pro-senescent [43]. However, it has subsequently been recognized that the senescent phenotype extends beyond the induction of growth arrest and encompasses functional traits that can alter the tumor microenvironment and tumor immunosurveillance [14]. Furthermore, the long-standing paradigm that senescence is an “irreversible” form of growth arrest has now been challenged by a growing collection of literature suggesting otherwise [113]. Our current understanding of the contribution(s) of senescence to cancer therapeutic outcomes are largely summarized below.

#### 2.2.1. Stability of the Senescent Growth Arrest

First, recent studies have provided compelling evidence that senescent tumor cells are in a “durable” but not permanent state of growth arrest, and that a subpopulation of tumor cells can and will ultimately recover proliferative capacity [24]. Early evidence in support of this was provided in studies by Elmore et al., wherein the evolution of senescence-resistant clones of MCF-7 breast tumor cells after exposure to Adriamycin suggested that the senescent growth arrest can potentially be evaded [114]. Then, Roberson et al. demonstrated the escape from TIS in H1299 non-small lung cancer cell model which lacks p53 and p16^INK4a^ functions [39]. It was subsequently shown that a variety of tumor cells exposed to genotoxic chemotherapy or ionizing radiation, with or without PARP inhibition (including lung, breast and colon), can escape from an established senescence phenotype, and resume growth, accompanied by partial or complete resolution of SA-β-gal staining and decreased expression of CDKIs (e.g., p21^Cip1^) and SASP factors (e.g., IL-1, IL-6, IL-8) [26,48]. To obviate concerns that recovery may be from non-senescent populations, work from our lab has directly observed the spontaneous division of senescent cells via live-cell imaging and high speed live cell interferometry [26,115]. Flow cytometry-based enrichment of SA-β-gal-positive (TIS) tumor cells implanted in immune-competent or immune-deficient animals further showed that senescent tumor cells can form viable tumors [26]. Similar results were obtained in a model of acute myeloid leukemia (AML), in which cytosine arabinoside promoted an escapable form of senescence in AML cells. Enrichment of these senescent cells and subsequent engraftment into mice was permissive for in vivo leukemia repopulation, suggesting that the recovery of senescent tumor cells may be sufficient for recurrent disease [56].

While the precise mechanism for escape from TIS is likely dependent on tumor type and therapy, a few mechanisms have been described thus far. For example, in p53-null lung cancers, overexpression of Cdk-2 was sufficient to bypass TIS growth arrest [39]. Downregulation of cell-membrane receptors for senescence-reinforcing proteins that are secreted during senescence may also be permissive for escape [116]. Finally, the ability to revert from senescence may also be attributed, at least in part, to large-scale gene expression alterations that allow for acquisition of stem cell-like characteristics, e.g., by activating the WNT signaling cascade [52,117]. This simultaneously allows for both proliferative recovery and the development of aggressive phenotypes (e.g., advanced migratory and invasive features) [51]. While the TIS response in tumor cells can be highly heterogenous [118], and although the ability of senescent cells to escape the growth arrest in patient tumors is still unknown, these cell-autonomous features of TIS support the strategies of eliminating senescent tumor cells using senolytics [64].

#### 2.2.2. Impacts on the Immune System

An additional, and rather severe, limitation to our understanding of how senescence influences the efficacy of therapy and patient outcomes relates to the interaction(s) between senescent tumor cells and the immune system. The ability of senescent cells to recruit immune cells appears to be largely non-cell autonomous and mediated through the SASP [57,119]. It is important to reiterate, however, that the SASP is highly heterogeneous, in part, related to the nature of the senescence-inducing stimuli and cell type [18]. Nevertheless, there are several factors that seem to be generally common, including the pro-inflammatory factors IL-6, IL-8, IL-1Β, and GROα [119]. Activation of the SASP has been directly linked to immune-mediated senescent cell clearance. For example, endometrial stromal cells that enter senescence during decidualization secrete IL-15 and are recognized and killed by uterine natural killer (NK) cells [120]. Liver cells that undergo oncogene-mediated senescence are recognized and eliminated by CD4^+^ T-cells [121], and senescent hepatic stellate cells have been shown to regulate and enhance activity of M1 macrophages [122]. Recruitment of immune cells by the SASP can also create further therapeutic vulnerabilities to immune checkpoint blockade [123]. This was shown by Ruscetti et al. in a model of pancreatic cancer, where intratumoral accumulation of CD8^+^ T-cells following TIS conferred increased response to anti-PD-1 therapy [123]. Importantly, the milieu secreted by senescent cells, and therefore the subsequent effect on immune cells, can be modulated by p53 status [122,124,125], likely due to its role in establishing the senescent response. For example, in the study on hepatic stellate cells, p53 -activated cells entered senescence and showed elevated secretion of IL-6, ICAM-1, and IFNγ. p53-suppressed, proliferating cells showed elevated secretion of IL-3, IL-4, and IL-5. These profiles directly affected macrophage phenotype, with the former promoting M1 polarization and the latter favoring M2 [122]. Furthermore, murine liver carcinomas with restored p53 activity entered senescence, and were suppressed through increased inflammatory cytokine secretion and activation of NK cells, macrophages, and neutrophils [125]. Given these observations, it appears that senescence and the SASP likely favor immune system recognition and tumor cell killing, so long as senescent cells can be recognized.

It is intuitive that senescent cell accumulation during the natural aging process must be associated with the ability to escape immune surveillance. In elderly individuals, this may be due, at least in part, to the natural decline in the immune system, called immunosenescence [126]. During immunosenescence, immune cells display many of the canonical features of senescence, including elevated expression of cyclin dependent kinase inhibitors, increased SA-β-Gal, and development of the SASP [126]. Notably, activity and production of cytotoxic molecules also decreases [126]. This phenomenon has repeatedly been linked to the higher occurrence of malignancies (and other immune-regulated illnesses) in the elderly [127], and arguably, would allow for the persistence of senescent cells, both normal and cancerous. Further, tumor induced T-cell senescence has also been reported [128,129,130], and this has been shown to be an additional mechanism of immune-evasion. This may also impair T-cell activity against senescent tumor cells. Finally, there have been reports of direct suppression and evasion of otherwise-healthy (i.e., non-senescent) immune cells by senescent tumor cells. For example, in a mouse model of aging, senescent stromal cells showed enhanced secretion of IL-6 as part of their SASP, and this resulted in increased levels of suppressive myeloid cells and the creation of a tumor-permissive microenvironment [131]. PTEN-deficient, senescent prostate cancer cells can also escape detection by secreting immunosuppressive factors, although this can be reversed by inhibiting STAT3/JAK signaling [132]. Senescent dermal fibroblasts have been shown to upregulate HLA-E, which interacts with an inhibitory receptor on NK cells and cytotoxic CD8^+^ T-cells [133]. High expression of matrix metalloproteases (often components of the SASP) can also cleave ligands for NKG2D receptors, allowing senescent cells to “shed” the signals that would otherwise mark the cells for NK-cell killing [134]. As such, whether or not the immune system efficiently recognizes TIS cancer cells is unclear and likely contextual. Overall, however, these observations suggest that at least a subpopulation of senescent tumor cells may eventually escape immunosurveillance, which could facilitate tumor recurrence.

#### 2.2.3. TIS in Non-Malignant Cells

It is further necessary to recognize that senescence induction in response to cancer therapy is not limited to tumor cells, as non-malignant cells can and do undergo senescence in response to chemotherapy or radiation. In fact, the induction of TIS in non-malignant cells has been linked to organ dysfunction involving the heart, kidneys, bone, bone marrow and nervous system, which all contribute to and/or may be central for many adverse effects of cancer therapy [55,135]. The persistence of these cells for prolonged periods, and their secretory ability, can drive an insidious inflammatory response that alters the homeostatic microenvironment [136]. Furthermore, as indicated in a previous section, the selective removal of these senescent cells, genetically or pharmacologically, was shown to result in beneficial outcomes. This was clearly demonstrated by the contribution of senescent cells accumulation to radiation-induced pulmonary fibrosis [88] or radiation-induced skin ulceration [80], both common complications of the use of radiotherapy for cancer.

#### 2.2.4. Evidence Supporting the Removal of Non-Malignant Therapy-Induced Senescent Cells

The cumulative evidence for deleterious consequences of TIS suggests that eliminating senescent cells is likely to prove beneficial, although, as indicated below, the clinical feasibility of this strategy remains to be unequivocally established. In 2017, Demaria et al. reported that the clearance of non-malignant senescent cells following doxorubicin treatment, in studies utilizing either a transgenic (p16-3MR) mouse model or the Bcl-2 family-targeting agent, navitoclax, resulted in less severe bone-marrow suppression, a decrease in cardiac dysfunction, a reduction in the development of metastases, and interference with tumor recurrence [55]. Another study has shown that elimination of senescent-like dorsal root ganglia neurons by navitoclax alleviated both mechanical and thermal hypersensitivity in a mouse model of cisplatin-induced peripheral neuropathy [137]. Similarly, using the INK-ATTAC model, doxorubicin-induced trabecular and cortical bone loss was alleviated by navitoclax, despite the absence of any evidence for a reduction in senescent cell mass in fat [135]. Collectively, this preclinical evidence appears to support the inclusion of senolytics as a component of cancer therapeutics, both to reduce tumor cell mass by the killing of senescent tumor cells, and by collaterally suppressing elements of normal tissue injury derived from the accumulation of non-malignant senescent cells [138]. This can be further encouraged, if senolytics were applied in a temporary fashion (separate pulses following the completion of chemotherapy treatment) in order to cull the burden, instead of the complete eradication, of senescent cells, in a manner that will avoid prolonged use of senolytics. However, in contrast to the hoped-for improvement in the efficacy of chemotherapy coupled with senolytics, the induction of TIS, of itself, was reported to improve outcomes in a cyclophosphamide-treated BCL-2 lymphoma mouse model; it should be noted, though, that evidence for senescence induction was extrapolated indirectly based on the functional status of the *INK4a* locus in tumor cells [139]. Moreover, senescence induction in colorectal cancer patients in response to 5-fluoruracil was correlated with higher progression-free survival, despite limited sample size [140]; similarly, daunorubicin- and cytarabine-induced senescence in acute myeloid leukemia patients was associated with better disease-free and overall survival states [141]. These in vivo findings coupled with the pre-clinical evidence clearly indicates that we still have an incomplete understanding of the nature of the contribution of senescence to overall cancer treatment outcomes, and whether senescence is ultimately a favorable tumor response to treatment.

### 2.3. Early Evidence on Senolytics as Anti-Cancer Therapies

By far, the most successful senolytic in preclinical cancer models has been navitoclax. Navitoclax has shown a remarkable capacity to eliminate tumor cells induced into senescence by a variety of therapies, including lung and melanoma cells treated with aurora kinase inhibitors or etoposide [142]; lung cancer cells treated with etoposide or radiation [27]; breast cancer cells treated with doxorubicin, radiation, or BET inhibitors [27,143,144]; ovarian cancer cells treated with PARP inhibitors [50]; and prostate tumor cells treated with PARP inhibitors or radiation [145]. Clearance of senescent tumor cells by navitoclax has been reported to enhance tumor regression/control and increase mouse survival [27,50,143]. As in non-tumor models, the senolytic activity of ABT-263 appears to depend upon inhibition of BCL-X_L_ [27,50]. Further, our laboratory has recently reported that BAX, but not BAK, is required for cell killing by ABT-263 [27]. However, in a recent publication with irradiated soft-tissue sarcoma, BCL-2 inhibition, albeit using supraclinical concentrations of ABT-199, was sufficient to promote senolysis, demonstrating the complex heterogeneity in which BCL-2 family member proteins senescent tumor cells depend upon for survival [146].

Despite the clear effectiveness of navitoclax in pre-clinical studies, navitoclax’s senolytic activity can be highly variable. For example, while prostate cancer LNCaP cells treated with antiandrogens enter senescence, the senescent cells are not eliminated by ABT-263 [145,147]; however, when treated with PARP inhibitors or radiation, the prostate tumor cells are, in fact, sensitive to senolytic action [145]. The authors have postulated that ABT-263 may only be effective in TIS systems associated with DNA-damage, a provocative concept that will require further studies for confirmation [145].

Another potential drawback to the use of senolytics such as navitoclax is that sensitivity to navitoclax is highly dependent on the expression levels of various BCL-2 family members, which can vary even within cancer cells of the same tumor type undergoing treatment with an identical therapy. This mechanistic element was highlighted by Shahbandi et al., whose work demonstrated that certain breast cancer cell lines remained insensitive to navitoclax following doxorubicin-induced senescence due to low levels of NOXA, a BH3-only protein that can neutralize the anti-apoptotic protein MCL-1 [143]. We have also observed this variability in lung cancer cell line sensitivity to navitoclax upon the promotion of senescence (unpublished data). Finally, the utilization of navitoclax in the clinic may be limited due to the development of severe thrombocytopenia in patients [148]. This toxicity appears to be a consequence of on-target BCL-X_L_ inhibition in platelets [148]. Because navitoclax’s senolytic activity is (often) dependent on BCL-X_L_, this **toxicity** appears to be unavoidable without advanced strategies to deliver the drug only to malignant cells. In addition to its known hematologic adverse reactions, navitoclax, when used at effectively senolytic doses, has been shown to cause detrimental bone changes such as decreased trabecular bone volume fraction of mice and impaired osteoblast function in vitro and in vivo, adding another layer of complexity for the use of navitoclax as a senolytic [149]. Its noteworthy that the last discussed evidence is from a single report, where similar adverse effects were not remarkably reported in clinical trials evaluating navitoclax. Lastly, the discussed evidence is largely based on short-term studies of these drugs as senolytics, overlooking any potential toxicities that can arise following chronic use of senolytics.

Although it might be tempting to propose the use of alternative senolytics in the clinic, the cancer field has yet to provide unequivocal pre-clinical support for any senolytic as an adjuvant therapy. There is far too little literature relating to the ability of other compounds, such as D+Q, piperlongumine, and fisetin to act as senolytics in the context of chemotherapy-induced senescence, and what is currently available is not particularly promising. For example, as opposed to its success in the context of aging-related processes, D+Q failed to kill senescent liver cancer cells, and each drug alone failed to kill senescent ovarian cancer cells [50,150]. Piperlongumine, while showing modest senolytic activity in olaparib-treated ovarian cancer [50], failed to kill senescent prostate cells [145]. Fisetin failed to kill senescent ovarian cancer cells [50], and, in our hands, was also largely ineffective against senescent lung, head and neck, and prostate cancer cells (unpublished data). Thus, similar to navitoclax, the senolytic activity of these agents may prove to be highly dependent on the cell lineage and senescence-inducing agents used.

The senolytic activity of a number of other agents that have been identified in tumor models, such as cardiac glycosides [151,152] and the HDAC inhibitor panobinostat [153] must also be considered with reservations. While cardiac glycosides have indeed been shown to eliminate senescent tumor and non-tumor cells, with claims of “broad-spectrum” activity, unfortunately these studies utilized supraphysiological concentrations of these agents that would pose intolerable toxicity to patients [151,152]. Also, there has been only a single report of panobinostat acting as a senolytic in head and neck and lung cancer cell lines induced into senescence by either cisplatin or taxol [153]. Interestingly, and perhaps unexpectedly, panobinostat was also shown, at certain concentrations, to promote senescence when administered in combination with cisplatin or taxol [153].

The HSP90 inhibitor 17-DMAG [61] and the BET protein family degrader ARV825 [154] have both shown broad spectrum activity across senolytic models, including TIS in cancer cells. However, data on these agents is limited, and HSP90 inhibitors can induce intolerable toxicity [155]. BET degraders, while presumably less toxic than BET inhibitors and seemingly “safe” in mice, have yet to be tested in humans [156]. It is possible, however, that the application of these drugs as senolytics may permit the use of lower doses, thereby reducing adverse events. Intriguingly, although ARV825 was proposed to induce senolysis in an autophagy-dependent manner [154], inhibition of autophagy has also been shown to promote senescent cell killing, due to elevated dependence on autophagy caused by proteotoxic stress [157]. Overall, taken together, the currently available evidence is consistent with the **lack of universality** for any individual senolytic amongst different therapy-induced senescent tumor cell models.

Another potential limitation to the utilization of senolytics is an apparent **resistance** of some senescent cell subpopulations. For example, while navitoclax has been shown to eliminate approximately 90% of SA-β-gal positive lung and breast tumor cells, there did appear to be residual surviving tumor cells that could reflect a resistant subpopulation [27]. While repetitive exposure to navitoclax (or other senolytics) might prove to eliminate these cells, possible heterogeneity of the senescent phenotype even within a monoclonally derived tumor cell population might confer resistance to senolysis. This analysis is based on data derived from studies in replicative exhaustion-induced senescent fibroblasts, where several distinct transcriptomic profiles can be identified within the same cell line associated with different phenotypic cell behaviors [20,21]. Post-senolysis, senolytic-resistant senescent cells could contribute to the proliferative recovery of a tumor cell population, over time, following the removal of a senolytic agent [27]. In all cases, the persistence of post-senolytic senescent cells is expected if senolytics are to be utilized in pulses to cull senescent cells, rather than their full eradication.

Finally, it has long been recognized that senescent cells contribute to a number of fundamental homeostatic processes. For example, senescence is a major tumor suppressor mechanism that halts the transformation of cells harboring carcinogenic mutations or oncogene overexpression [22,158]. So long as senolytics kill senescent cells, rather than hinder the ability to enter the senescent growth arrest, there is little concern that they might be permissive for malignant transformation. Furthermore, although oncogene-induced senescence may be tumor suppressive by one interpretation, accumulation of either oncogene-induced or natural aging related senescence may promote and/or support tumorigenesis through the SASP [159]. This notion is supported by studies from Baker et al., in which aged INK-ATTAC mice that were cleared of p16-positive cells showed increased latency to lethal cancer development [160]. As such, elimination of oncogene- or aging-induced senescent cells are likely of little concern to patient health and would certainly be in-line with senolytic efforts to mitigate aging-related pathological processes. On the other hand, embryonal senescent cells play an important role during organogenesis through paracrine communication with differentiating cells [161]. It is not certain whether these cells persist or carry other physiological roles during adult life. It is also not certain whether they will inadvertently be eliminated by senolytics, especially in that these variants do not show DNA damage or depend on classical cell cycle regulators for their growth arrest. Senescent cells also accumulate at wound sites and participate in the acute inflammatory reaction that mediates wound healing. Moreover, the removal of p16^INK4a^-positive cells from active wounds using the p16-3MR model interferes with the healing process [162]. This suggests that these cells might also be eliminated in parallel with senescent tumor cells and could result in unwanted impairment of wound healing. Lastly, senescence is involved in recruiting a T cell-mediated immune response against certain viral infections [163], and **non-selective** removal of these cells by senolytics can potentially increase the risk of viral replication. Overall, the inability of senolytics to differentiate between pathological and physiological senescent cells could represent an additional limitation to their use as cancer therapeutics.

**Figure 1:**
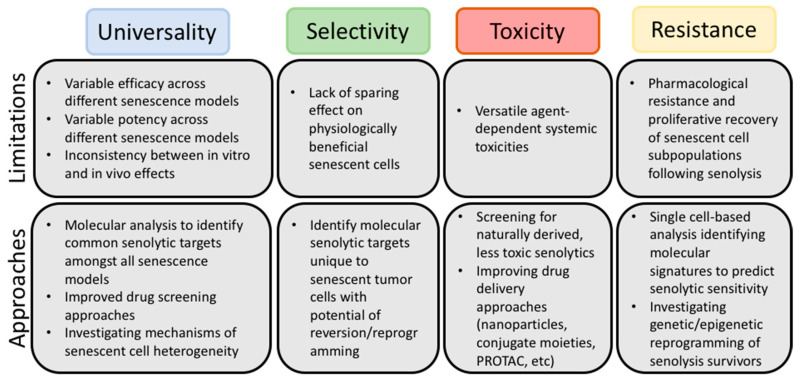
Limitations of current senolytics and potential approaches for circumvention. Although senolytic agents have shown promise as adjuvant cancer therapies preclinically, careful review of the literature has elucidated common limitations. The above figure outlines four outstanding issues with current senolytics, defines these issues, and summarizes the approaches that may mitigate or circumvent these issues, as outlined in detail in Section 3.

Given the current state of the literature, we have identified four significant challenges (Figure 1) that should be addressed as research continues: (i) the lack of universality of the current senolytics and the need for the identification of the conditions under which each agent is best used, (ii) the selectivity of senolytics towards “harmful”, rather than “beneficial”, senescent cells, (iii) strategies to avoid toxicity associated with the senolytics and (iv) identification of potential resistance mechanisms. In the following section, we will propose various approaches that could be implemented in order to optimize the translational success of multiple senolytic agents.

## 3. Possible Refinements of the Senolytic Strategy

Although the challenges we have thus far identified are certainly hindrances to the translation of senolytics in cancer therapy, they are not insurmountable. Indeed, efforts to optimize senolytic strategies have already been described in the literature, and there are experimental paths forward that should allow the precise, pragmatic application of senolytic therapies. Below, we discuss several approaches to be considered in future research.

### 3.1. Multi-Model Screenings

Our limited understanding of why some TIS models fail to respond to certain senolytics may be largely due to the absence of extensive multi-model approaches. While it is true that the identification of new senolytics has been based on screening multiple types of senescence-associated stimuli—e.g., replicative-, oncogene-, and stress-induced, often these studies do not involve tumor cell lines or anticancer therapies that differ markedly with regard to their mechanisms of action. For example, the senolytic ability of navitoclax was first identified in WI-38 fibroblasts induced into senescence by replicative exhaustion, oncogene overexpression and DNA damage precipitated by exposure to ionizing radiation [62]. Shortly afterwards, navitoclax senolytic activity was confirmed using irradiated HUVEC and IMR90 cells [84]. To their credit, the authors of these papers did establish that navitoclax may work in various “forms” of senescence, but the studies were limited to non-malignant cell lines. A similar approach (i.e., the use of non-malignant cell lines exposed to various forms of senescence-inducing stimuli) has been adopted in other screens for agents with senolytic activity; as a result, multiple senolytics have been identified that appear to work in non-tumor cell lines, but often fail when tested against TIS cancer cells (see Section 2.3). This observation strongly suggests that there are likely to be fundamental differences in the pathways that promote survival in senescence that occurs in tumor and non-tumor cells and argues for the inclusion of cancer cell lines for future senolytic screening efforts. The senolytics discussed here and the models in which they have been tested are summarized in Table 1.

The lack of universality of senolytics is also likely a consequence of both the genetic and phenotypic heterogeneity amongst different cancer/TIS models [15,118]. Bojko et al. have demonstrated that exposure of multiple tumor cell lines, specifically A549, SH-SY-5Y, HCT116, MDA-MB-231, and MCF-7 to different genotoxic chemotherapeutics including doxorubicin, irinotecan, methotrexate, 5-fluorouracil, oxaliplatin, and paclitaxel was associated with different degrees of senescence as well as differing senescence marker expression [118]. The extent to which senescence is induced, as well as the associated molecular manifestations, will arguably play a role in susceptibility to different classes of senolytic agents. A deeper understanding of what determines the senescence response to specific chemotherapies would likely facilitate being able to predict the likelihood of a patient’s tumor entering into senescence, and whether the utilization of a senolytic might be indicated; this, of course, is further handicapped by our limited understanding of senescence in vivo [166], and potential modulation of sensitivity to senolytics by tumor microenvironment [167].

The bulk of the published literature shows evidence of navitoclax’s senolytic activity in models where senescence was induced in tumor cells by genotoxic chemotherapy, which raises the possibility that DNA damage might be a prerequisite for ABT-263′s senolytic action [145]. However, navitoclax has also been shown to target senescent tumor cells induced by the CDK 4/6 inhibitor palbociclib, which is not generally associated with classical DNA damage [168]. Therefore, it remains unknown whether (and why) certain forms of TIS are inherently refractory to senolysis by navitoclax (or other agents). Thus, the diverse mechanisms whereby senescence-promoting therapy induces senescence in tumor cells must be taken into consideration upon performing wide drug screening, and an established representative of each drug class must be considered.

An ideal experimental approach for the development of future senolytic therapeutics might be to screen for senolytic activity across multiple cancer cell lines utilizing multiple relevant therapies. This will require wide-spectrum high throughput screening approaches, and of course, these studies will likely need to be stratified according to cancer type, where only the cancer-type relevant therapies are screened. However, the development of comprehensive profiles of senolytic function across multiple genotypes, with various cancer therapies, should ultimately provide a finely tuned delineation of specific senolytics that are effective against specific types of cancers, and in response to disease-relevant therapies.

### 3.2. Single Cell-Omics

The mRNA profiles of senescent cells are remarkably different from their proliferating or quiescent counterparts [13]; however, these senescence-associated transcriptional profiles are also heterogenous among senescent cells [15]. These variabilities create a formidable challenge in identifying biological markers of senescence in vivo. Accordingly, gene expression analysis studies have focused on identifying novel transcriptomic profiles commonly shared by most senescent cells generated in different models. These efforts have extended to the investigation of these variations at a single cell level within a common population of senescent cells in culture [12]. Using single-cell isolation and a nanofluidic PCR, Wiley et al. demonstrated a higher variability in *LMNB1* (encoding for Lamin B1) SASP-related gene expression in senescent IMR90 human fibroblasts and a more consistent expression of *CDKN1A* (encoding for p21^Cip1^) amongst individual cells. Chen et al. utilized single cell full-length RNA-seq to demonstrate six transcriptomic clusters and three distinct cell lineages within a common passage of senescent MEFs [20]. Interestingly, senescent cells exhibited variability in gene expression related to apoptosis regulatory pathways, some of which are the targets for the senolytic navitoclax. This is of particular relevance since, as noted previously, a subpopulation of senescent cells in culture may fail to respond to a single exposure to navitoclax. This apparent refractoriness to a senolytic could be explained, in part, by different expression profiles of the senolytic molecular targets in subpopulations of cells even within cells derived from a clonal lineage. This assumption is largely supported by Shahbandi et al.’s observations where certain doxorubicin-induced tumor cell lines were not initially sensitive to navitoclax and required further interference with other BCL-2 family members—namely, the concomitant inhibition of MCL-1, while other cell lines exposed to the same stimulus were readily susceptible to senolysis by navitoclax [116]. Consequently, utilizing these single cell-based technologies might explain the differential senolytic potential of navitoclax and other drugs within a single lineage of senescent cells growing in an identical environmental milieu and allow for a better understanding of the mechanisms of and resistance to senolysis. Unfortunately, these types of gene expression analyses have not, thus far, been performed in senescent tumor cells; this may be a practical consideration, in that, an exceptionally high variability might be anticipated in tumor cells that intrinsically have a high degree of genomic instability. Nevertheless, performing these types of analyses in TIS models could enable the identification of the cell variants resistant to senolysis in terms of their molecular phenotypes and contribution to the post-senolysis proliferative recovery often observed [27]. Furthermore, this approach could reveal universal senolytic molecular targets as well as facilitating the identification of novel senolytics. Importantly, such single cell analyses can, in fact, be performed in animal models, having been conducted in aging mice to identify senescence-associated genomic profiles that can be used to predict the effect of senolytics in vivo [169]. The ultimate goal of this approach would be to identify unique molecular signatures that would allow for the prediction as to whether a newly identified senolytic will display favorable effects in clearing senescent tumor cells in vitro and in vivo, especially if coupled with the multi-model strategy proposed above.

### 3.3. Improving Targeted Drug Delivery

As mentioned previously, many identified senolytics are accompanied by unfavorable toxicity profiles [148]. Furthermore, it is established that senescent cells are involved in homeostatic physiological processes including wound healing and embryonal development [161,162]. Although senolytics have been tested for potential adverse effects in mouse models, an examination of their effects on these senescence-specific functions has not received sufficient attention. Whether the systemic use of senolytics will inadvertently affect these homeostatic functions remains to be examined.

One strategy to potentially enhance the selectivity and mitigate the toxicity of senolytic compounds is through their structural modification. One such modification is a nanoparticle delivery system, in which navitoclax is nano-encapsulated in a galactose-coated particle [170]. The high levels of SA-β-gal in senescent cells should allow for enhanced release of navitoclax in senescent cells over non-senescent cells. Theoretically, this would lower the systemic toxicity of the drug, as healthy platelets should not be able to “digest” the nanoparticle. This “nanosenolytic” approach resulted in the killing of palbociclib-induced senescent triple negative breast cancer cells and led to decreased tumor volume and metastatic burden in vivo [170]. While the benefit of the nanosenolytic over navitoclax was only marginal with regards to effects on the tumor, the encapsulated form of navitoclax showed less toxicity as measured by both animal weight, survival, and platelet number [170]. Intriguingly, the galactose-coated nanoparticle approach may also allow non-senolytic agents to target senescent cells; for example, in a publication by Muñoz-Espín et al., galactose-coated nanoparticles encapsulating doxorubicin acted as a senolytic, preferentially killing palbociclib-induced senescent cells. In addition to clearing senescent tumor cells and reducing tumor burden in vivo, the doxorubicin nanoparticle produced less cardiotoxicity than free doxorubicin [171]. Given these data, nanoparticle delivery appears to be a promising approach to targeting senescent cells while eliminating or reducing systemic toxicity.

A similar approach for mitigating the toxicity of senolytics is their direct conjugation to galactose [168]. As with the nanoparticle system, galacto-conjugation of navitoclax showed selective senolytic activity in senescent lung cancer cells, provided almost equal benefit to free navitoclax in tumor control, but spared platelets both ex vivo and in vivo [168]. Thus, galacto-based modifications of navitoclax appears promising, at least in terms of preventing on-target toxicity in non-senescent cells and can likely be extended to alternative senolytic agents. Further, these types of particles will likely maintain senolytic activity in non-tumor, senescent cells, allowing for the clearance of senescent stroma. As previously discussed, this may further benefit patient outcome.

Still, “healthy” senescent cells like those involved in wound-healing may suffer in the presence of senolytics. To that end, the generation of proteolysis targeting chimera (PROTAC) agents appears to represent a viable alternative strategy [172,173]. PROTACS allow for greater selectivity than free senolytics due to the additional E3 ligase ligand moiety, which can be tailored for tissue and cell type. The first BCL-X_L_ PROTAC described was derived from the navitoclax structure and a ligand for the Von Hippel-Lindau (VHL) E3 ligase, which was found to be highly expressed across human tumor cells and minimally expressed in normal platelets [172]. This differential ligand expression afforded greater selectivity for BCL-X_L_-dependent tumor cells than navitoclax both in vitro and in vivo [172]. A second report more clearly defined the senolytic capacity of a BCL-X_L_-targeting PROTAC [173]. In this instance, the PROTAC was targeted to the E3 ligase cereblon; this bivalent molecule had potent senolytic activity in vitro—in some cells, more potently than navitoclax—and cleared senescent cells in aged mice without a significant reduction in platelet number [173]. While this study showed significant clearance of non-malignant senescent cells, the cereblon ligase was chosen based only on its low expression in platelets as opposed to other cell types. The choice of an E3 ligase that is preferentially expressed in cancer cells, such as VHL, conjugated to a senolytic moiety, may confer greater selectivity for senescent cancer cells over senescent, non-malignant cells. While the VHL PROTAC study did not directly address senescence and senolytic capacity of the PROTAC, it quite clearly demonstrated that the PROTAC synergized with senescence-inducing chemotherapies such as doxorubicin [172].

### 3.4. Identification of Novel Drug Targets that Selectively Eliminate “Bad” Senescent Cells

In addition to structural modifications that may spare “good” senescent cells, the field may very well be able to identify specific vulnerabilities or features of “bad” senescent cells that allow for their selective clearance. For example, only a subpopulation of senescent tumor cells can escape the stable growth arrest and recover proliferative capacity [26,39]. This escaping population is likely to account for the aggressive phenotypes that arise following recovery from senescence [51]. Accordingly, identifying targetable pathways unique to the senescent tumor cell subpopulation(s) with potential for resurgence might be of value. For example, the WNT signaling pathway is pivotal in facilitating the tumor-regenerating abilities of senescent tumor cells as part of a wide transcriptomic and functional reprogramming [52]; consequently, targeting elements of WNT signaling might serve as a selective senolytic approach that spares those senescent cell that have a lower probability to contribute to cancer recurrence.

**Figure 2:**
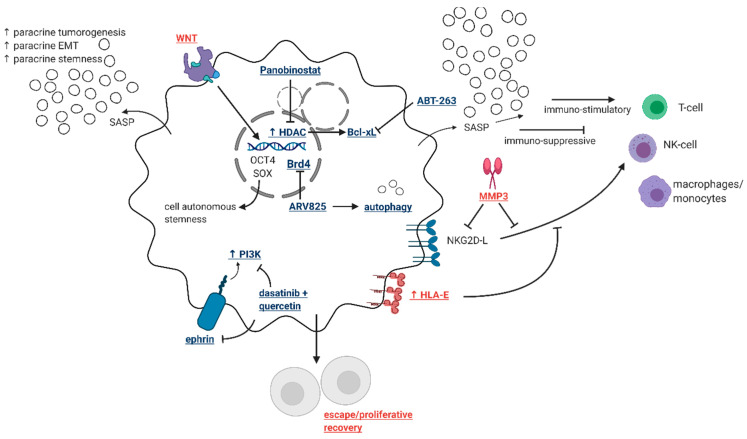
Validated and potential therapeutic targets to eliminate senescent cells. The accumulation of senescent cells is arguably disadvantageous to patient outcome due to the potential for proliferative recovery, the development of cell-autonomous stemness, SASP-driven stimulation of neighboring cells, and SASP-mediated immunogenicity. Despite evidence that SASP can drive immune-cell driven elimination of senescent cells, the SASP can also be strongly immunosuppressive. Further, elevated matrix-metalloprotease secretion and HLA-E expression have been demonstrated mechanisms by which senescent cells continue to evade immune cell detection. Multiple small molecule drugs have been identified and validated as senolytics (blue bolded text), but we propose that specific targeting of the more detrimental features of senescent cells (red bolded text) may prove efficacious alternatives or supplementations to the identified drug targets. This figure was generated through biorender.com.

Another unique aspect of senescence is that a certain fraction of senescent tumor cells becomes polyploid, which has been linked to the acquisition of stem cell-like characteristics, self-renewal and resistance to therapy [174,175]. Consequently, eliminating those polyploid senescent cells might be necessary to mitigate the unfavorable effects of therapy-induced senescence without interfering with other “useful” senescent populations [176]. These approaches and how they compare to established senolytics are illustrated in Figure 2.

It is apparent that certain senescent tumor cells are capable of evading immune recognition. This was delineated by Muñoz et al. who described the ability of some senescent tumor cells to shed surface immune markers—namely, NKG2D ligands, through the proteolytic action of matrix metalloproteases (MMPs) secreted as part of the SASP [134]. Loss of such membrane-bound ligands interferes with the ability of immune cells to recognize and eliminate senescent cells, and therefore targeting such a mechanism could also serve as a strategy to remove only those senescent cells with “bad intentions”. One final strategy to target senescent cells that may otherwise have evaded the immune system is chimeric antigen receptor T cell based senolytics [165]. As demonstrated by Amor et al., CAR T-cells can be engineered to attack senescence-specific cell membrane proteins that are not typical immune-regulatory antigens, leading to T-cell-mediated clearance of senescent tumor cells both in vitro and in vivo [165].

## 4. Conclusions

There is little question that senolytic agents constitute a promising addition to conventional and possibly also targeted cancer therapies that promote tumor cell senescence. It is now largely accepted that senescence is likely to be an undesirable outcome of cancer therapy in terms of the detrimental effects of the secretions from senescent cells as well as the potential of senescent tumor cells to escape from arrest and regenerate the disease. However, there is limited information available as to whether senescence is actually a central response to therapy in the clinic, either in the primary tumor or in residual surviving tumor cells, despite extensive evidence for this outcome in preclinical experimental model systems. Other issues that remain to be resolved include the lack of uniformity in the action of senolytics against aging related pathologies and tumor cell senescence, durability of the response, the development of resistance and toxicity to normal tissue. Nevertheless, there do appear to be a variety of strategies available for circumventing issues of toxicity, including structural modifications and drug delivery systems. Consequently, it is likely that a more in-depth understanding of the factors that determine which “types” of senescence are susceptible to the senolytics will ultimately result in these agents being incorporated into standard of care, at least for certain types of cancers and in combination with select antitumor drugs.

## Figures and Tables

**Table 1:** The most commonly utilized senolytics in pre-clinical models. A multitude of compounds with differing mechanisms of action of been described as senolytics. This table, although not comprehensive of all such agents, summarizes the most commonly tested compounds, their mechanism(s) of action, and the models in which they have been tested for senolytic activity regardless of their success in exerting senolysis.

Senolytic	Mechanism	Model	Reference(s)
**Dasatinib + Quercetin**	Dasatinib: tyrosine kinase inhibitor Quercetin: flavanol that inhibits various kinases, including PI3K	Irradiated preadipocytes, HUVEC cells, and MEFS; chronic atherosclerotic vascular disease; radiation- or bleomycin-induced lung fibrosis; aging-associated hepatic steatosis; arteriovenous fisulation in chronic kidney disease; Alzheimer’s disease; hyperoxia-induced airway dysfunction; obesity-associated anxiety; obesity-induced metabolic dysfunction; osteoarthritis; radiation-induced bone degeneration; radiation-induced ulceration; doxorubicin-treated HepG2 and Huh-7 cells	[60,68,69,70,71,72,73,74,75,76,77,78,79,80,81,150]
**Navitoclax (ABT-263)**	Inhibits BCL-2, BCL-X_L_, and BCL-W	Radiation-treated HUVEC, IMR90, and MEF cells; Irradiated bone marrow stem cells; Aging associated bone loss; radiation-induced and bleomycin-induced lung fibrosis; lung emphysema; utrine leiomyoma; tau-dependent neurogeneration; radiation-induced neurodegeneration; myocardial infarction; heart failure; pulmonary hypertension; insulin resistance; osteoarthritis; syntehetic implant-mediated fibrosis; Duchenne muscular dystrophy; doxorubicin-treated and MDA-MB-231 cells; doxorubicin-treated SKBR7, Cal 51, 4226, HCC712, MDA-MB-175, MCF-7, HCC1428, ZR75-30, T47D, U2OS, and MPE600 cells; etoposide-treated and irradiated A549 cells, PARPi-treated OV1369(R2), OV90, OV4453, and OV1946 cells; PARPi-treated and irradiated LNCaP and PC3 cancer cells; irradiated STS93, STS109, and STS117 cells.	[27,50,62,84,87,88,89,90,91,92,93,94,95,96,97,98,99,100,101,143,145]
**Panobinostat**	HDAC inhibitor	Cisplatin and taxol treated A549, H460, H1355, FaDu, UMSCC47, and UMSCC1 cells	[153]
**17-DMAG**	HSP-90 inhibitor	Oxidative stress induced senescent MEFs and MSCs; etoposide-treated IMR90 cells; telomere-shortening induced senescent WI38 cells	[61]
**Piperlongumine**	Multi-faceted; potentially via inhibition of oxidation resistance 1 protein	Irradiated, RAS-overexpressing, and replication-induced senescent WI-38 cells; PARPi-treated OV1369(R2), OV90, OV4453, and OV1946 cells; enzalutamide-treated LNCaP cancer cells	[50,107,145,164]
**Curcumin/EF-24**	Unidentified; proteasomal degradation of MCL-1 and BCL-X_L_	Senescent intervertebral disc cells; irradiated and replication-induced senescent WI-38, IMR-90, HUVEC, HREC, and preadipocyte cells	[108,109]
**Fisetin**	Unidentified	Oxidative-stressed induced MEFs; etoposide-treated or irradiated IMR90 cells; irradiated HUVECs; irradiated primary human preadipocytes; progeroid mice; PARPi-treated OV1369(R2), OV90, OV4453, and OV1946 cells	[50,105,106]
**Cardiac** **glycosides**	Inhibit Na^+^/K^+^ ATPase pump; increase expression of NOXA	Bleomycin-, gemcitabine-, doxorubicin-, etoposide-, and palbociclib-treated A549 cells; palbociclib-treated SK-MEL-103; RAS-overexpressing and H202-treated senescent primary BJ cells; senescent HaCat, H1299, U373-MG, H1755, and MCF-7 cells; osteoarthritic chondrocytes; breast cancer PDXs; lung fibrosis; RAS-overexpressing, replicative-induced, and etoposide-, doxorubicin-, and palbociclib-treated IMR90 cells; primary bronchial epithelial cells;	[151,152]
**Chimeric** **antigen receptor (CAR) T cells**	T cells engineered to target specific cell membrane proteins such as urokinase-type plasminogen activator receptor to redirect specificity to senescent cells	MEKi/CDK4/6i-treated KP cells; hepatic NRAS^G12V^-expressing NSG mice; murine liver fibrosis	[165]
**Bromodomain and extra-terminal (BET) family protein inhibitors/degraders**	Prevent DNA repair and increase autophagy via degradation of the BET protein BRD4	RAS-overexpressing IMR90 cells; replication-induced, RAS-overexpressing, and doxorubicin-treated TIG-3 cells; obesity-induced murine hepatocellular carcinoma; doxorubicin-treated HCT116 cells;	[154]

Cell lines: WI-38: human diploid lung fibroblasts. IMR90: fetal lung fibroblast. MEFs: mouse embryonic fibroblasts. MCS: mesenchymal stem cells. HUVEC: human umbilical vein endothelial cells. HREC: human retinal endothelial cells. BJ: human fibroblasts. HaCat: human keratinocytes. TIG-3: fetal lung fibroblast. HEPG2 and Huh7: liver cancer cells. MDA-MB-231, MDA-MB-175, SKBR7, Cal-51, HCC712, MCF7, HCC1428, ZR75, T47D, 4226, MPE600: breast cancer. U2OS: osteosarcoma. A549, H460, KP, H1335, H1299, H1755: lung cancer. OV1369(R2), OV90, OV4453, and OV1946: ovarian cancer. FaDu, UMSCC47, UMSCC1: head and neck cancer. LNCaP, PC3: prostate cancer. SK-MEL-103: melanoma. HCT116: colorectal cancer. U373-MG: glioblastoma. STS93, STS109, and STS117: soft tissue sarcoma.
